# Electro-Magnetism a Remedy for Opium Poisoning

**Published:** 1843-08

**Authors:** E. E. Marcy

**Affiliations:** Hartford


*Electro-Magnetism a Remedy for Opium Poisoning.* By E. E. Marcy, M.D., Hartford, July 3d, 1843.—A few weeks since I was called upon to visit the child of Mr. H. Poster, of this city, who had taken an over-dose of laudanum. On entering the room I found the child (eight months of age) in an apparently dying state. The pulse was almost entirely imperceptible at the wrist; respiration suspended, except at long intervals; pupils not contracting to the light; the extremities cold, and an entire insensibility to external impressions. From the history of the case it was ascertained that about two teaspoonsful of the narcotic had been administered by a girl with whom the child had been left in charge, some eight or ten hours previously, and during the absence of the mother.

From the apparent hopelessness of the case, I was deterred from making use of the ordinary means of resuscitation, being fully aware that the poison had entered the system, and was working its fatal effects on the brain, &c.

The respiration and circulation were nearly suspended, and unless some means could be taken to remove the death-like torpor of the brain, it was evident that these vital functions would cease to be executed. I therefore at once resolved to make use of a powerful electro-magnetic machine, hoping that I might arouse and maintain the action of the *brain* sufficient to keep up *respiration* and *circulation* until the effects of the laudanum should pass off.

First, a smart shock was passed through the head with no apparent effect—then six or eight were passed in rapid succession, when slight convulsive motions were perceived. By continuing the shocks at short intervals for thirty minutes, the child was restored sufficiently to open its eyes and take some notice of things when presented to him—the respiration had also been constantly improving, until it had now become natural—the pulse had also acquired strength and regularity, and the temperature of the body become natural.

The shocks were now discontinued for a time, when the stupor, with gasping respiration and thready pulse, &c., returned. The use of the machine again, like magic, restored the patient to life and consciousness.

The remedy was persevered in until the effects of the poison had passed off, and the child was restored to safety.

It must be borne in mind that opium destroys life by suspending for a time the function of the brain, and thus secondarily putting a stop to *respiration* and *circulation*, which are dependent on the brain for their action.

In all those cases, therefore, where the narcotic has been absorbed into the system, we believe the only sure way of proceeding is to make those applications which shall act directly in *arousing the action of the brain*, and thus keep in operation the vital organs which are dependent on it, until the stupor has disappeared. The most effectual agent for accomplishing this is, without doubt, the electro-magnetic machine.—*Boston Med. and Surg. Journ.*, July 12, 1843.

